# Vegetation biomass and topography are associated with seasonal habitat selection and fall translocation behavior in Arctic hares

**DOI:** 10.1007/s00442-024-05534-x

**Published:** 2024-03-30

**Authors:** Ludovic Landry-Ducharme, Sandra Lai, François Vézina, Andrew Tam, Dominique Berteaux

**Affiliations:** 1 Département de Biologie, Chimie et Géographie, Université du Québec À Rimouski, Rimouski, QC Canada; 2 Canada Research Chair On Northern Biodiversity, Université du Québec À Rimouski, Rimouski, QC Canada; 3 Centre for Northern Studies, Université du Québec À Rimouski, Rimouski, QC Canada; 4 Quebec Centre for Biodiversity Science, Université du Québec À Rimouski, Rimouski, QC Canada; 5 Department of National Defence, 8 Wing Canadian Forces Base Trenton, Astra, ON Canada

**Keywords:** Herbivore, *Lepus arcticus*, Migration, Stopover, Tundra

## Abstract

Habitat selection theory suggests that environmental features selected at coarse scales reveal fundamental factors affecting animal fitness. When these factors vary across seasons, they may lead to large-scale movements, including long-distance seasonal migrations. We analyzed the seasonal habitat selection of 25 satellite-tracked Arctic hares from a population on Ellesmere Island (Nunavut, Canada) that relocated over 100 km in the fall. Since no other lagomorph is known to perform such extensive movements, this population offered an ideal setting to test animal movement and habitat selection theory. On summer grounds hares selected low elevation areas, while on winter grounds they selected high vegetation biomass, high elevation, and steep slopes. During fall relocation, they alternated between stopover and traveling behavioral states (ratio 2:1). Stopover locations were characterized by higher vegetation heterogeneity and lower rugosity than traveling locations, while vegetation biomass and elevation interacted to explain stopover locations in a more complex way. The selected combination of environmental features thus varied across seasons and behavioral states, in a way broadly consistent with predictions based on the changing food and safety needs of hares. Although causality was not demonstrated, our results improve our understanding of long-distance movements and habitat selection in Arctic hares, as well as herbivore ecology in the polar desert. Results also provide strong support to animal movement and habitat selection theory, by showing how some important hypotheses hold when tested in a species phylogenetically distinct from most animal models used in this research field.

**Supplementary Information:**

The online version contains supplementary material available at 10.1007/s00442-024-05534-x.

## Introduction

Animals move to change the environmental conditions associated with their location (Van Moorter et al. 2016). A general understanding of movement behavior thus requires studying location changes from the perspective of the animal in its environment, that is habitat selection. When an animal selects a specific set of conditions influencing its fitness, it seeks the best balance between the factors limiting its survival and reproduction (Northrup et al. 2022). Since such combinations vary in space and time, habitat selection underlies movements at various spatial and temporal scales, depending on individual needs and the extent of landscape heterogeneity. Habitat selection may thus underly decisions ranging from modifications of daily activity (Roberts et al. 2017) and movements within home ranges (Gedir et al. 2020), to home range shifts (Nakashima et al. 2013) and seasonal migrations (Hebblewhite & Merrill 2009).

Habitat selection has commonly been approached as a nested top-down process (Paton & Matthiopoulos 2016) since Johnson (1980) identified four orders of selection ranging from the species’ distribution (order 1) to microhabitats (order 4). In complement, Rettie & Messier (2000) proposed the limiting factor avoidance hypothesis, where the most limiting factors act at broad spatial and temporal scales and constrain selection at finer scales, thus inducing responses of animals at all these scales. According to this hierarchical framework, habitat selection studies conducted at broad spatiotemporal scales may reveal processes of primary ecological importance, among which is seasonality.

Seasons generate major variations in external (e.g., availability of food resources, predation risk, competition intensity) and internal (e.g., reproductive state, body condition) factors affecting fitness, thus modifying their relative importance to habitat selection (Godvik et al. 2009; McLoughlin et al. 2011). Not surprisingly, selected habitats can thus change across seasons, sometimes entailing broad-scale movements (Courtemanch et al. 2017; Hebblewhite & Merrill 2009). Accordingly, many species inhabiting highly seasonal ecosystems, such as alpine and polar environments, undertake large-scale seasonal migrations (Teitelbaum et al. 2015). This is the case of most Arctic breeding birds, which migrate to temperate regions in winter to benefit from more abundant resources and milder climate (Somveille et al. 2015), and many caribou populations (*Rangifer tarandus*) which migrate between calving and winter grounds to track variations in forage quality and predation risk (Gunn et al. 2009).

Among mammalian terrestrial herbivores, the longest seasonal movements are found in large species such as ungulates (Avgar et al. 2013; Gnanadesikan et al. 2017). Likely, the physiological constraints of energy deposition and the high energetic cost of terrestrial locomotion in small mammals (Hedenström, 2003) explain their inability to undertake long-distance movements (Hein et al. 2012). As a result, research on large-scale movements of terrestrial mammals is strongly biased towards ungulates (Berteaux & Lai 2021; Gnanadesikan et al. 2017), despite the importance of varying study models across taxonomic groups to test ecological hypotheses.

The Arctic hare (*Lepus arcticus*) is one of very few herbivores living year-round in the High Arctic polar desert. This ecosystem is characterized by very low winter temperatures (down to − 40 °C), low summer temperatures (often around 0 °C), very low primary production (< 5% plant cover, 2-month growing season), and strong seasonal contrasts in light availability (Bliss 2000). Persistence of homeotherms in this environment is a considerable biological challenge. Given their limited ability to store energy, Arctic hares mostly rely on their high mobility to exploit local food patches and avoid predation (Klein & Bay 1994). Arctic hares are generalist herbivores, feeding on woody plants, sedges, lichens, and grasses (Best & Henry 1994; Klein & Bay 1994; Schaefer et al. 1996). They seem to prefer broken terrain and sidehills, which facilitate escape from predators and offer easier access to vegetation where snow is blown by the wind (Aniskowicz et al. 1990; Best & Henry 1994; Gray 1993). In general, mating occurs in April–May, leverets are born in June, and mothers raise them alone and nurse them until August (Best & Henry 1994). As for many other small mammals (Schweiger et al. 2021), information on Arctic hare habitat selection and movement ecology is scarce and mostly anecdotal (Best & Henry 1994). Some populations display short seasonal range shifts (Hearn et al. 1987; Small et al. 1991), but Arctic hares were until recently considered largely resident, like all other lagomorphs. However, Caron-Carrier et al. (2022) reported large-scale (ca. 100 km), synchronized and directional fall movements of Arctic hares on northeastern Ellesmere Island (Canada). These movements could be part of a seasonal migration triggered by the large-scale resource heterogeneity of the polar desert (Caron-Carrier et al. 2022; Lai et al. 2022), but determining Arctic hare habitat selection across seasons is required to better understand the drivers of this outstanding behavior.

In this context, our general objective was to assess the influence of vegetation and topography on habitat selection by Arctic hares on northeastern Ellesmere Island, across three critical phases of their annual space use strategy, namely summer residency when leverets are born and raised, winter residency when meteorological conditions are most constraining, and fall relocation when many individuals travel from summer to winter grounds. Our general hypothesis was that two main habitat components (plant biomass providing food, and topographical features decreasing predation risk) are selected, but differentially across the annual cycle depending on energy needs and predation vulnerability. Specifically, we hypothesized that summer habitat selection is driven by the minimization of predation risk (H1), given that females raise vulnerable leverets. We thus predicted that hares select summer habitats with highly heterogeneous relief offering numerous hiding and escaping opportunities (P1). Second, we hypothesized that winter habitat selection is mostly driven by the maximization of energy gains, and secondarily by the minimization of predation risk (H2). We thus predicted that in winter, hares select habitats with the highest plant biomass (P2a), as well as steep slopes such as wind-swept mountain sides (P2b), which give easier access to vegetation while also facilitating escape from predators. Third, we hypothesized that during fall relocation hares need to stop to replenish their energy reserves given their limited fuel storage capacity, which should structure habitat selection depending on behavioral state (H3). We thus predicted that relocating hares alternate between stopover episodes characterized by limited and non-directional movements, and traveling episodes characterized by longer and more directional movements (P3a), and that habitats selected for stopovers have a higher plant biomass than those selected to travel (P3b).

## Materials and methods

### Study area

**Fig. 1:**
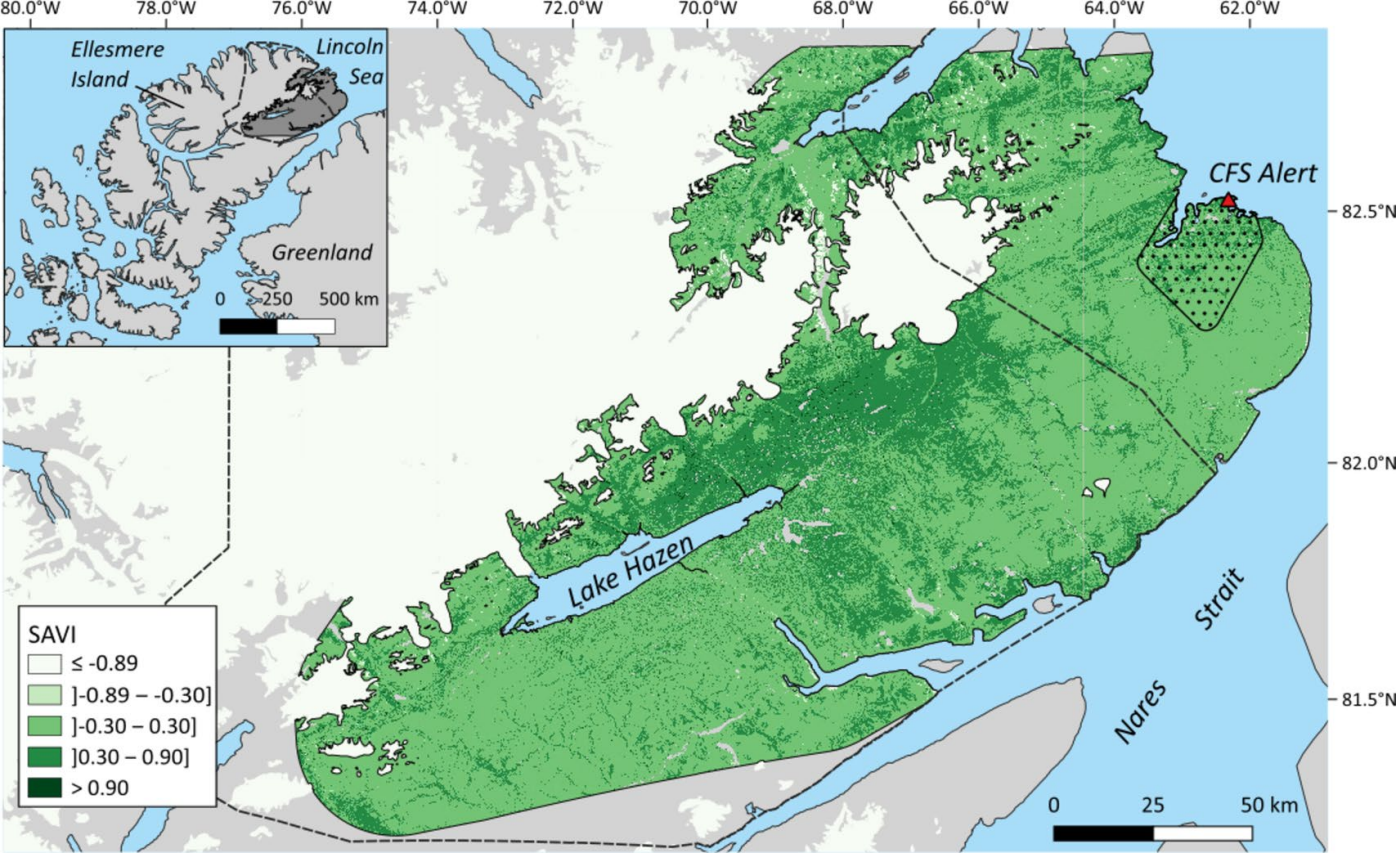
Location of the study area on Ellesmere Island (Nunavut, Canada). Insert shows part of the Canadian Arctic Archipelago and Greenland, with the study area (dark grey) being enlarged on the right. Winter (shades of green within solid line) and summer (black dots within solid line) availability areas were estimated from locations of Arctic hares tracked from June 2019-May 2020. Major non-habitat features such as large waterbodies (blue) and glaciers (white) were excluded from seasonal availability areas. The green color shades indicate classes of vegetation biomass as reflected by the soil-adjusted vegetation index (SAVI). Canadian Forces Station Alert is located at the northeastern tip of the area (red triangle). The dashed line indicates boundaries of Quttinirpaaq National Park of Canada. This map was created using QGIS 3.16.16 (QGIS.org 2022). Projection of the map is WGS 84/Pseudo-Mercator (EPSG 3857)

We worked at the Canadian Forces Station (CFS) Alert (82° 30’ N, 62° 20’ W), on the northeastern tip of Ellesmere Island (Nunavut, Canada) (Fig. 1). Due to animal movements, the 19,250 km^2^ study area extends south of CFS Alert to encompass most of Quttinirpaaq National Park of Canada and the lands extending east of the park (Fig. 1). The region is part of the polar desert biome (Bliss 2000). Plant communities mostly consist of herbaceous and low shrub species, mosses, and lichens (Desjardins et al. 2021a, 2021b; Government of Canada 2018). The area surrounding CFS Alert is rugged with hills, small lakes, and ponds. The annual frost-free period lasts 28 days and the average annual precipitation is 156 mm (Government of Canada 2013). The area north-east of Lake Hazen in Quttinirpaaq National Park of Canada (Fig. 1) is a thermal oasis standing out from the surrounding barren plateaus (France 1993; Government of Canada 2018). It is characterized by a less rugged terrain and a milder and damper microclimate, with numerous wet tundra meadows which favor plant and animal diversity. Common terrestrial wildlife in the study area includes, in addition to the Arctic hare, muskox (*Ovibos moschatus*), Peary caribou (*Rangifer tarandus pearyi*), collared lemming (*Dicrostonyx groenlandicus*), and rock ptarmigan (*Lagopus muta*) (Government of Canada 2013, 2018). The main terrestrial predator of adult hares is the Arctic wolf (*Canis lupus arctos*) (Mech 2007), while juveniles are also vulnerable to the Arctic fox (*Vulpes lagopus*), ermine (*Mustela erminea arctica*), and snowy owl (*Bubo scandiacus*) (Dalerum et al. 2017; Government of Canada 2013; Hearn et al. 1987).

### Capture and tracking of hares

In June˗July 2019, we captured Arctic hares around CFS Alert using Tomahawk cage traps (102 cm × 38 cm × 38 cm, model 208, Tomahawk Live Trap Co., Tomahawk, WI, USA) and custom-made drop cages (95 cm × 95 cm × 45 cm). We used peanuts and commercial bird food as bait and inspected cages every 2–4 h. Upon capture, we ear-tagged hares using custom-made plastic color tags (1.5 cm × 2.5 cm) attached to metal bands (Jiffy Wing Bands – Style 893 Size 4, National Band and Tag Co.). We fitted 25 hares with a collar carrying an Argos Platform Terminal Transmitter (model KiwiSat 303, Lotek, Newmarket, Ontario, Canada; 115 g or 2–3.1% of body mass). Transmitters were programmed to obtain fixes from 10:00 to 13:00 local time daily for at least 12 months. The repetition rate was set at 60 s and multiple locations were usually obtained per 3-h period. Capture and handling techniques were approved by the Animal Care Committee of Université du Québec à Rimouski (CAC-68-17-184) and the Government of Nunavut (WL 2018-020). Our sample size consisted of 21 females, including 17 that were confirmed breeders, and 4 males (details in Caron-Carrier et al. 2022). The dataset generated and analyzed for this study is part of the Arctic Animal Movement Archive (Davidson et al. 2020), is freely available in MoveBank (Wikelski et al. 2021), and is stored in the MoveBank Data Repository (Berteaux 2021). We filtered the dataset based on a 5 km/h cruising speed threshold (with possible acceleration bouts of 10 km/h for locations less than 10 min apart) using a speed filter implemented in R (version 4.1.1; R Core Team, 2021). We used locations with Argos location classes 1 (1500-m isotropic error), 2 (500 m), 3 (250 m), and A (no error estimation) (Christin et al. 2015), and kept one location per day per individual based on the smallest error obtained (Caron-Carrier et al. 2022).

### Environmental variables

Based on our predictions, we tested the effect of 11 environmental variables on hare habitat selection. Variables describe vegetation biomass, elevation, slope, rugosity, and aspect (Table 1). We built a vegetation map of the study area using Google Earth Engine (Gorelick et al. 2017) from the Sentinel-2 MultiSpectral Intrument level 2 dataset available in the Google Earth Engine’s public data archive. We filtered, based on a 5% cloud criterion, Sentinel-2 images acquired daily from 15˗30-Jul-2019, the peak period of the Normalized Difference Vegetation Index (NDVI). We masked permanent waterbodies using the CANVEC 50k Nunavut database (Natural Resources Canada 2019). From the collection of filtered images, we created a single median image, from which we calculated the Soil-Adjusted Vegetation Index (SAVI) (Huete 1988), which adjusts NDVI for areas with low plant cover by minimizing the influence of variations in soil brightness.

**Table 1:** Environmental variables used to analyze Arctic hare seasonal habitat selection on northeastern Ellesmere Island (Nunavut, Canada) in 2019–2020

Variable	Description	References
SAVI_mean	Mean value of soil-adjusted vegetation index (estimates average plant biomass)	Aniskowicz et al. (1990);Best & Henry (1994); Dalerum et al. (2017); Klein & Bay (1994); Schaefer et al. (1996); Small et al. (1991)
SAVI_SD	Standard deviation of soil-adjusted vegetation index (estimates spatial heterogeneity in plant biomass)	Idem
Elevation_mean	Mean value of elevation (m)	Aniskowicz et al. (1990); Best & Henry, (1994); Mercer et al. (1981); Schaefer et al. (1996)
Slope_mean	Mean value of slope (degrees)	Idem
Rugosity	Arc-chord ratio (ARC) rugosity index (estimates spatial heterogeneity in elevation)	Du Preez (2015)
North*	Proportion of area covered by North-facing slopes (315–45°)	Aniskowicz et al. (1990); Best & Henry (1994); Gray (1993); Mercer et al. (1981)
East*	Proportion of area covered by East-facing slopes (45–135°)	Idem
South*	Proportion of area covered by South-facing slopes (135–225°)	Idem
West*	Proportion of area covered by West-facing slopes (225–315°)	Idem
Northness**	Relation to North (1 = North, -1 = South)	Idem
Eastness**	Relation to East (1 = East, -1 = West)	Idem

Variables were extracted at a 10-m spatial resolution and are continuous. Aspect variables used only during hare residency are identified with a star while those used only during hare relocation are identified with two stars. References provide a biological justification for the choice of variables. All variables were standardized prior to statistical analyses

In QGIS 3.16.16 (QGIS.org 2022), we computed topographic features from the ArcticDEM 2-m resolution mosaic (Porter et al. 2018) accessed through the Google Earth Engine’s public data archive. We resampled rasters at a 10-m resolution to match the SAVI raster resolution (Vaze et al. 2010). We used the elevation raster to derive elevation, slope angle, aspect and the arc-chord ratio (ACR) rugosity index (Du Preez 2015). For slope aspect, we classified aspect values as North (315–45°), East (45–135°), South (135–225°), and West (225–315°) oriented slopes.

All spatial data were analyzed using the WGS 84/Arctic Polar Stereographic projection.

### Habitat selection during residency

#### (a) Determination of home ranges and availability areas

We determined each phase of the seasonal cycle (summer residency, winter residency, fall relocation) for each individual based on their average daily movement rates (details in Caron-Carrier et al. 2022). Locations of resident hares were included in the summer residency dataset if collected before the date of departure of the last relocating hare. The winter residency dataset included locations of hares which left Alert in fall and established a distinct winter range.

We adopted a use-availability design to investigate habitat selection at the regional scale (order 2 of Johnson (1980)) during summer and winter residency. Following the study design 2 of Manly et al. (2007), we defined, separately for each season, habitats used at the individual scale (home ranges) and habitats available at the population scale. We estimated home ranges using the 100% adaptative-LoCoH method (Getz et al. 2007) implemented in the R package “adehabitatHR” (version 0.4.19; Calenge 2006). This nonparametric kernel method considers holes and boundaries attributable to geomorphologic features such as lakes, cliffs, or shores. Based on the area-observation curve displaying variation of home range size according to number of locations, we kept for analysis only individuals with enough locations (n > 50) to reach a stable home range. We delineated availability areas (Fig. 1) separately for summer and winter because the extent of locations differed across seasons.

For summer, we considered as available the area included in a 100% Minimum Convex Polygon (MCP) (Mohr 1947) calculated from summer locations of all individuals, to which we added a buffer of 2.6 km, corresponding to the radius of the mean individual 100% summer MCP. We masked non-habitat features (major waterbodies and glaciers) using CANVEC 50K Nunavut hydrographic features shapefile (Natural Resources Canada 2019) in QGIS 3.16.16. To compare used-to-available habitats, we created random home ranges by randomly moving and rotating each individual home range across the defined availability area. We employed a used:available home range ratio of 1:5, which was visually considered adequate for the 475-km^2^ available area.

We proceeded similarly to define the winter availability area. However, the added buffer was 24.5 km and the used:available home range ratio was 1:10 given the considerably larger winter availability area (19,250 km^2^). Table S1 (Online Resource) shows variability of environmental features within summer and winter availability areas.

#### (b) Habitat selection in summer and winter

We estimated habitat-selection functions for each season separately using logistic regression with binomial distribution and logit link function. We did not use mixed-effect models since the dataset included only one home range per individual (no intra-individual variance). We extracted from observed and randomly generated home ranges the mean and standard deviation of SAVI, the mean elevation, rugosity, the mean slope, and the proportion of home range covered by each aspect category. We standardized covariates (Schielzeth 2010) to facilitate subsequent model convergence and interpretation of estimates. We kept only the most biologically relevant variable for sets of variables with Spearman rank correlation > 0.7. Variables retained for habitat selection analyses appear in Table S2 (Online Resource) for each season.

We selected models based on AICc (Burnham & Anderson 2002) using R package “MuMIn” (Barton 2020). Models were ranked based on AICc values and AICc weights of evidence (wi), considering models with ΔAICc < 2 as having substantial empirical support (Burnham & Anderson 2002). Among these models, we assessed uninformative parameters by checking if the 85% confidence intervals of coefficients overlapped 0, keeping for further interpretation only models with informative variables (Arnold 2010; Harrison et al. 2018). We chose 85% confidence intervals (rather than 95%) because AIC-based model selection keeps variables when their approximately 85% confidence intervals excludes zero (Arnold 2010). We validated model assumptions using R packages “performance” (version 0.8.0; Lüdecke et al. 2021) and “DHARMa” (version 0.4.3; Hartig 2021). We assessed multicollinearity by ensuring that the variance inflation factor (VIF; Zuur et al. 2010) never exceeded 5 (R package "car"; Fox & Weisberg 2018). A variable’s regression coefficient being the logarithm of the relative selection strength (RSS; Avgar et al. 2017), we calculated and plotted the log-transformed RSS using R package “amt” (version 0.1.7; Signer et al. 2019).

### Habitat selection during relocation

According to stopover ecology theory (Evans & Bearhop 2022; Hedenström, 2003), we expected movement patterns of hares relocating between summer and winter habitats to show a traveling state (locations distant from each other and roughly aligned) and a stopover state (locations clustered). We identified these two behavioral states using a path segmentation analysis based on hidden Markov models (HMM), which allows detection of underlying behavioral processes (Edelhoff et al. 2016). We first regularized trajectories with missing locations to 1 location/day by interpolation (mean = 9.3% of an individual’s locations; range 0–26.3%). We then fitted the HMM using a gamma state-dependent distribution to model step lengths, and a wrapped Cauchy distribution to model step angles. To ensure numerical stability, we ran ten iterations choosing initial parameters randomly within a plausible range according to the behavioral state (Grenier-Potvin et al. 2021). We graphically assessed model’s goodness-of-fit by ensuring that the distribution of pseudo-residuals did not significantly deviate from a normal distribution. We finally used the Viterbi algorithm with the resulting model to assign the most likely state to each location. We used the R package “MoveHMM” (version 1.7; Michelot et al. 2016) to prepare data, fit HMM, and classify locations.

To compare environmental features selected by hares at stopover sites and in traveling areas, we elaborated a latent selection difference analysis (Mueller et al. 2004) by fitting a mixed-effect logistic regression with binomial distribution and logit link function, including random intercept among individuals (Gillies et al. 2006; Viejou et al. 2018). The stopover state was considered as outcome (1) and the traveling state as exposure (0). Provided 95% confidence intervals do not overlap zero, coefficients of this analysis indicate the relative selection of a covariate for one state compared to the other. Positive values indicate stronger selection in the stopover state compared to the traveling state. To consider the uncertainty of Argos locations and the ability of hares to perceive their surroundings, habitat variables were calculated in a buffer around locations. The radius of the buffer matched the Argos estimated error of the location (Argos class 1 = 1,500 m, 2 = 500 m, 3 = 250 m). Buffers around A-class Argos locations, which have no accuracy estimate, were assigned a 1500-m radius (see Fig. S1 in Online Resource for the distribution of error classes associated to locations according to each behavioral state). We extracted the mean and standard deviation of SAVI, mean elevation and mean slope, for all buffered stopover and traveling locations, and values were standardized (Table S3 in Online Resource). The sizes of buffered locations were too small to calculate aspect class proportions, thus we created continuous aspect variables by trigonometrically transforming aspect to represent its relations to north and east (northness and eastness; Zar 2010). We extracted the mean values of northness and eastness for each buffered location. We considered two-term interactions between mean SAVI and mean elevation. We selected models and verified assumptions as previously described for habitat-selection functions. We assessed models’ goodness-of-fit by calculating the area under the curve (AUC) of a bootstrap receiver operating characteristic (ROC) curve with 999 repetitions (Hosmer et al. 2013) using R package “ROCR” (version 1.0–11; Sing et al. 2005).

Results are expressed as mean ± SD.

## Results

### Habitat selection during residency

#### (a) Home ranges

We tracked 25 individuals during summer (21 females, 4 males), for a total of 1174 filtered locations (47 ± 24 locations per individual) obtained from 15-Jun-2019 to 17-Sep- 2019. For the summer habitat selection analyses, we considered the 11 individuals for which there were enough locations to obtain a stable home range estimate (70 ± 10 locations per individual, Table S4 in Online Resource). Summer home ranges measured 10.4 ± 4.7 km^2^ (range: 5.0–19.5 km^2^, *n* = 11). Additional movement metrics such as timing, duration, and distance of relocation are reported in Caron-Carrier et al. (2022).

A total of 21 hares relocated to winter grounds, and 20 settled in a winter range (1 died during relocation), for a total of 1669 filtered locations (79 ± 61 locations per individual) obtained from 10-Sep-2019 to 31-May-2020. Mortality (*n* = 18) and battery failure (*n* = 2) throughout winter resulted in 11 individuals with sufficient locations to be included in the winter habitat-selection analysis (122 ± 62 locations per individual) (Table S4 in Online Resource). Winter home range measured 91.7 ± 79.9 km^2^ (range: 23.2–281.7 km^2^, *n* = 11). Three individuals had enough locations in both summer and winter to be included in both seasonal analyses.

#### (b) Habitat selection during summer

**Fig. 2:**
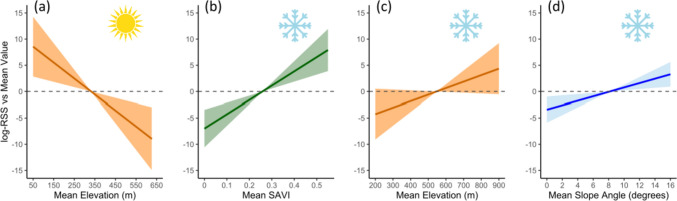
Representation of variables included in the best model of regional-scale habitat selection analyses for an Arctic hare population studied in summer (**a**) and winter (**b**–**d**) on northeastern Ellesmere Island (Nunavut, Canada). Slopes show variation in the logarithm of the relative selection strength (log-RSS) between value of the variable in a home range and mean value of the variable among home ranges of all individuals included in the analyses. RSS quantifies the difference in probability of selection between two locations differing in one environmental variable (other variables being held constant), thus allowing for standardized comparison of effect sizes. Positive values indicate selection and negative values indicate avoidance. In summer (sun symbol), hares select relatively low-elevation home ranges (**a**). In winter (snowflake symbol), they select home ranges relatively rich in vegetation (**b**) and high in elevation (**c**), and with relatively steeper slopes (**d**). Confidence intervals were estimated using the standard errors of the models. SAVI stands for soil-adjusted vegetation index

Mean elevation was included in all models with ΔAICc < 2 (Table 2 in Online Resource). The most parsimonious model only included a strong negative effect of mean elevation on summer home range selection (log-RSS = − 4.43; CI 95% [− 8.14–− 2.20]). Holding all other variables constant, the probability of home range selection by hares was divided by 20 for each elevation increase of 100 m (Fig. 2a). Vegetation biomass was not selected in summer at the studied spatial scale. Contrary to P1, factors reflecting relief heterogeneity were not selected.

**Table 2:** Comparison of the best models (ΔAICc < 2), the full model, and the null model in an analysis of regional-scale habitat selection of Arctic hares tracked on northeastern Ellesmere Island (Nunavut, Canada) during summer 2019

Model (summer)	K	− LL	ΔAICc	W
Elevation_mean + North	3	15.35	0	0.23
Elevation_mean + East	3	15.51	0.33	0.20
Elevation_mean + North + East	4	14.59	0.62	0.17
**Elevation_mean**	**2**	**17.06**	**1.33**	**0.12**
Elevation_mean + South + East	4	15.13	1.7	0.10
Elevation_mean + SAVI_mean + North	4	15.17	1.78	0.10
Elevation_mean + North + South	4	15.26	1.96	0.09
SAVI_mean + Elevation_mean + Rugosity + North + East + South (Full)	7	14.57	7.24	0.01
Null	1	36.86	38.86	0.00

The retained model (bold) was the most parsimonious one. We give for each model the number of parameters (K), the negative log-likelihood (− LL), the difference in Akaike’s information criterion adjusted for small sample size (AICc) compared to the model with the lowest AICc (ΔAICc), and the AICc weight of evidence (w). See Table 1 for description of variables

#### (c) Habitat selection during winter

The most parsimonious model included mean vegetation biomass, mean elevation, and mean slope (Table 3), in accordance with P2a and P2b. Vegetation biomass was omnipresent in concurrent models, being the most influential variable in the best model (log-RSS = 3.40; CI 95% [2.01–5.71]) (Fig. 2b). The probability of home range selection was multiplied by 15 for each increase of 0.1 in mean SAVI in the home range (P2a supported). Mean elevation was also found in all concurrent models, having (unlike in summer) a positive effect on selection (log-RSS = 2.02; CI 95% [0.29–5.01]) (Fig. 2c). The probability of home range selection by hares was multiplied by 3.4 for each 100-m increase in home range mean elevation. Mean slope also had a positive effect on home range selection (log-RSS = 1.57; CI 95% [0.55–2.91]) (Fig. 2d), resulting in a probability of selection multiplied by 1.5 for each additional degree of the mean slope in the home range (P2b supported).

**Table 3:** Comparison of the best models (ΔAICc < 2), the full model, and the null model in an analysis of regional-scale habitat selection of Arctic hares tracked on northeastern Ellesmere Island (Nunavut, Canada) during winter 2019–2020

Model (winter)	K	− LL	ΔAICc	w
**SAVI_mean + Elevation_mean + slope_mean**	**4**	**13.95**	**0**	**0.21**
SAVI_mean + SAVI_SD + Elevation_mean + South	5	13.11	0.48	0.16
SAVI_mean + SAVI_SD + Elevation_mean	4	14.31	0.72	0.14
SAVI_mean + Elevation_mean + Slope_mean + South	5	13.35	0.97	0.13
SAVI_mean + Elevation_mean + Slope_mean + North	5	13.55	1.37	0.10
SAVI_mean + SAVI_SD + Elevation_mean + North	5	13.71	1.69	0.09
SAVI_mean + Elevation_mean + Slope_mean + West	5	13.78	1.82	0.08
SAVI_mean + SAVI_SD + Elevation_mean + Slope_mean	5	13.86	1.98	0.08
SAVI_mean + SAVI_SD + Elevation_mean + Slope_mean + North + South + West (Full)	8	12.93	6.89	0.01
Null	1	36.86	39.5	0.00

The retained model (bold) was the most parsimonious one. We give for each model the number of parameters (K), the negative log-likelihood (− LL), the difference in Akaike’s information criterion adjusted for small sample size (AICc) compared to the model with the lowest AICc (ΔAICc), and the AICc weight of evidence (w). See Table 1 for description of variables

### Habitat selection during relocation

**Fig. 3:**
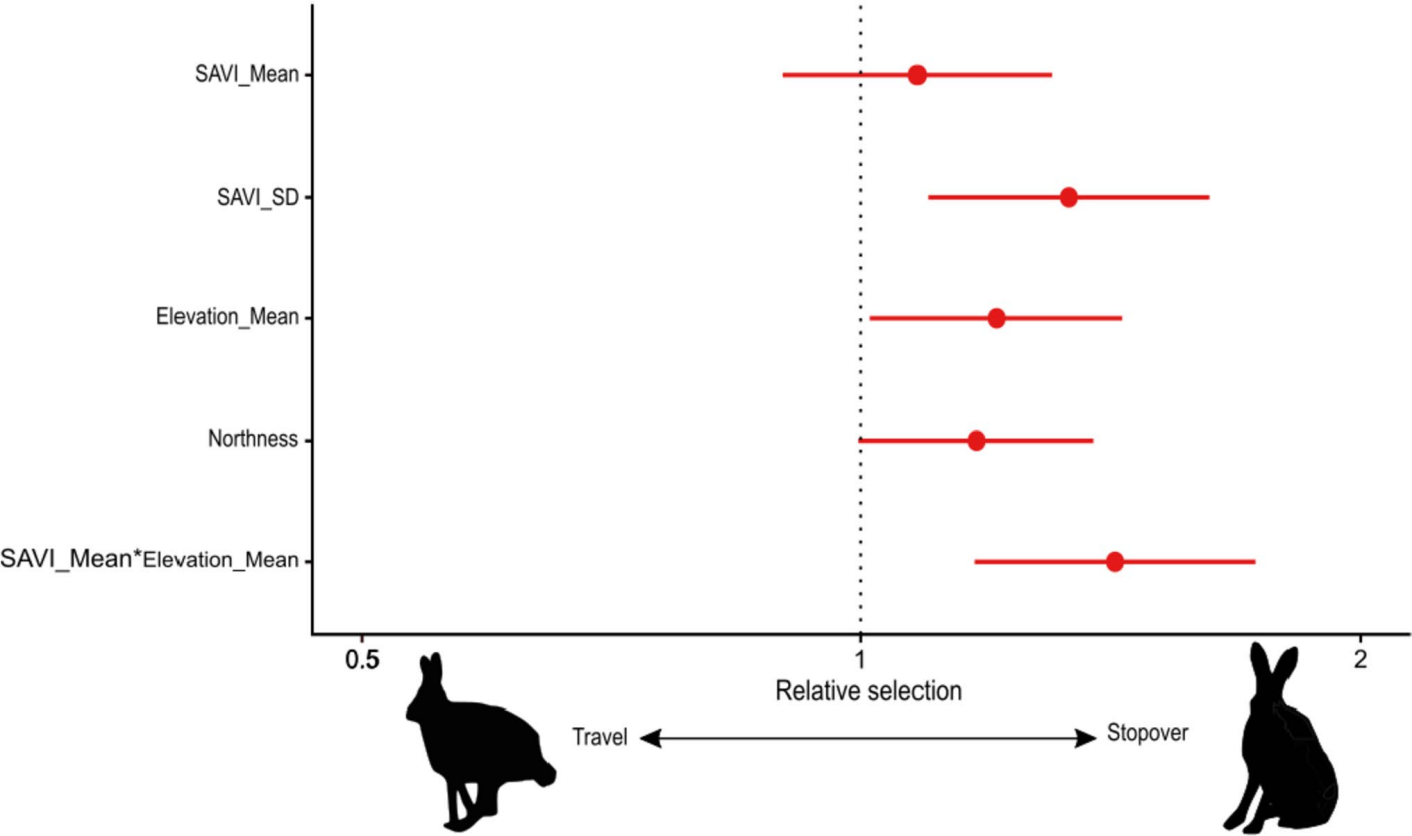
Odds ratio and 95% confidence interval of variables included in the best model of a latent selection difference analysis of stopover and traveling buffered locations during the 2019 fall relocation of 21 Arctic hares on northeastern Ellesmere Island (Nunavut, Canada), with stopover state as outcome. Positive values indicate stronger selection in the stopover state compared to the traveling state. Confidence intervals overlapping 1 indicate no substantial difference between states. SAVI stands for soil-adjusted vegetation index. Northness is a continuous aspect variable created by trigonometrically transforming aspect to represent its relations to north (1 = North, − 1 = South, see Methods)

**Fig. 4:**
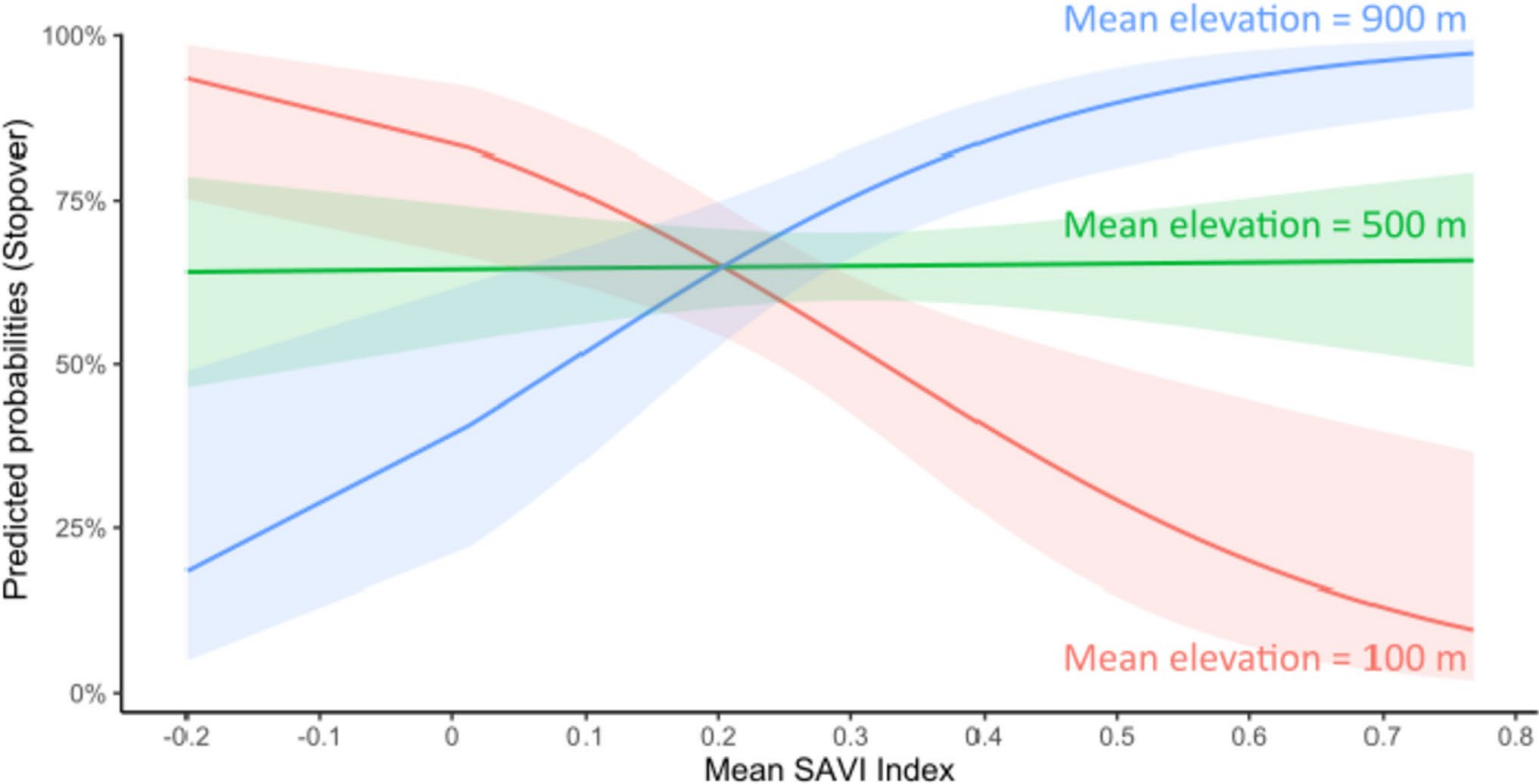
Representation of the interaction between mean soil-adjusted vegetation index (SAVI) and mean elevation in a latent selection difference analysis of stopover and traveling buffered locations during the 2019 fall relocation of 21 Arctic hares on northeastern Ellesmere Island (Nunavut, Canada). When hares were at high elevation (900 m, blue), their relative probability of stopping was highest when SAVI was high, but when they were at low-elevation (100 m, red), their relative probability of stopping was highest when SAVI was low. SAVI had no effect on the relative probability of stopping when hares were at intermediate elevation (500 m, green). Shaded areas represent 95% confidence intervals

The 21 relocating individuals yielded 793 locations obtained from 11-Aug-2019 to 5-Nov-2019. These locations could be categorized as stopover and traveling locations by the path segmentation analysis, supporting P3a. The resulting two-state model (AIC = 7014.5) fitted data better than a one-state model used as control (AIC = 7329.6). About twice as many locations were classified as stopover compared to traveling locations, but substantial variation existed among individuals (Table S5 in Online Resource). In partial support to P3b, the most parsimonious model describing stopover sites included mean vegetation biomass in interaction with mean elevation (Fig. 3; Table 4). The probability of stopping was the greatest where both elevation and vegetation biomass were highest, or conversely where both elevation and vegetation biomass were lowest (P3b partially supported; Fig. 4; Table S6 in Online Resource). At intermediate elevations, the probability of stopping did not depend on vegetation biomass. In addition, an increase of 0.1 of the standard deviation of vegetation biomass around a location multiplied by 1.46 the probability of stopping, suggesting that hares stopped preferentially in patches of heterogeneous vegetation. Although present in our best model, there was only weak evidence that northness affected the probability of a location to be a stopover, since the lower limit of the confidence interval was very close to zero for both variables (Fig. 3; Table S6 in Online Resource). Model performance assessment with ROC attested that our model had some reasonable predictive power (AUC = 0.62 ± 0.04).

**Table 4:** Comparison of the best models (ΔAICc < 2), the full model, and the null model in a latent selection difference analysis of stopover and traveling buffered locations during the 2019 fall relocation of Arctic hares on northeastern Ellesmere Island (Nunavut, Canada)

Model (fall)	K	− LL	ΔAICc	w
SAVI_mean + SAVI_SD + Elevation_mean + Northness + Eastness + SAVI_mean*Elevation_mean + (1 | ID)	8	494.70	0	0.34
SAVI_mean + SAVI_SD + Elevation_mean + Northness + SAVI_mean*Elevation_mean + (1 | ID)	**7**	**496.20**	**0.96**	**0.21**
SAVI_mean + SAVI_SD + Elevation_mean + Eastness + SAVI_mean*Elevation_mean + (1 | ID)	7	496.68	1.92	0.13
SAVI_mean + SAVI_SD + Elevation_mean + Slope_mean + Northness + Eastness + SAVI_mean*Elevation_mean + (1 | ID) (Full)	9	494.44	1.53	0.16
Null	1	516.13	929.96	0.00

The retained model (bold) was the most parsimonious one. We give for each model the number of parameters (K), the negative log-likelihood (− LL), the difference in Akaike’s information criterion adjusted for small sample size (AICc) compared to the model with the lowest AICc (ΔAICc), and the AICc weight of evidence (w). See Table 1 for description of variables

## Discussion

Our study is the first to address Arctic hare habitat selection through a use-availability design, and also the first to address habitat selection in a lagomorph population showing large-scale seasonal movements. In accordance with our general hypothesis, plant biomass and topography influenced hare habitat selection at the regional scale, and their relative importance varied across seasons. Also confirming predictions, hares alternated between stopover and traveling states when relocating during the fall, and again plant biomass and topography influenced the location of these behaviors. The only strong discrepancy between predictions and observations was that summer habitat selection was explained by terrain elevation rather than terrain rugosity. As detailed below, our results broadly corroborate animal movement and habitat selection theories, hint to a scale-related limitation in our summer study design, and open stimulating research avenues regarding Arctic hare behavior, terrestrial mammal migrations, and polar desert ecology.

### Movements and habitat selection of herbivores across seasons

#### (a) Home range size

A key determinant of herbivore home range size is food availability, so that populations living in poor habitats compensate with larger home ranges (McLoughlin & Ferguson 2000). Habitat productivity decreases with latitude, thus it is not surprising that Arctic hare home range sizes on northern Ellesmere, at the northern limit of the species range, were 6 (summer) to 80 times (winter) larger than those described at the southern limit in Newfoundland (Hearn et al. 1987). Methodological differences in data collection and home range estimation technique may account for some of these latitudinal discrepancies. Nonetheless, as showed by Nilsen et al. (2008), such large variations much more likely arise from ecological differences between study sites.

#### (b) Seasonality of food and safety needs

In several northern and alpine herbivores, seasonality affects the relative influence of food resources and predation risk on habitat selection. For example, moose (*Alces alces*) show weaker selection in winter than any other season for patches near roads (which facilitate predator movements), regardless of their quality (McLoughlin et al. 2011). Likewise, red deer (*Cervus elaphus*) selection for cover habitat is higher in summer and winter, when movements are impeded respectively by calves and snow, than in fall and spring, when emphasis is on food intake (Godvik et al. 2009). Accordingly, our results show different effects of plant biomass and topographical features on habitat selection of Arctic hares in summer and winter, confirming suggestions by Dalerum et al. (2017) and Small et al. (1991).

We had predicted (P1) that heterogeneous relief would be the main factor influencing summer habitat selection of adult females (which dominate our sample) but rather observed elevation to be the main factor. We are, however, hesitant to reject the hypothesis (H1) that summer habitat selection is driven by the minimization of predation risk for three non-exclusive reasons. First, the spatial scale at which our study takes place probably captured poorly the fine-scale ruggedness created by boulder fields, which may be what females selected to rest or safely nurse their leverets. Second, boulder fields mostly occurred at low elevations, generating a spatial correlation between elevation and fine-scale ruggedness that potentially precluded detection of relief effects. Finally, our sample sizes were rather small (11 home ranges in both summer and winter), which may have precluded statistical detection of some relationships. This combination of elements reminds us how difficult it is to design observational studies that are suited to understand habitat selection at all relevant scales (McGarigal et al. 2016; Schweiger et al. 2021).

In contrast, our results confirmed that hares select winter habitats with the highest plant biomass (P2a) and with relief features favoring accessibility to forage while reducing predation risk (P2b). These results are in good agreement with Parker's (1977) field observations on nearby Axel Heiberg Island. Food resources may thus be the main limiting factor driving wintering hares to the regionally rich basin north of Lake Hazen (Fig. 1), thus explaining their surprising (for a lagomorph) fall relocation. In addition to a limited sample size, one important aspect to consider during result interpretation, however, is the absence of information on predators. Other herbivores, such as caribou and muskoxen, aggregate in winter around Lake Hazen, therefore wolves also likely do so (France 1993; Government of Canada 2018). Predators can either track prey abundance when prey are aggregated, track prey habitat when prey are uniformly distributed, or track prey catchability when prey are difficult to catch (Zabihi-Seissan et al. 2022). No information allows us to interpret winter habitat selection of hares around Lake Hazen in relation to these alternatives.

### Migration and stopover ecology of running mammals

The limited fuel storage capacity of small mammals limits the distance that they can cover without refueling, thus favoring winter adaptations other than migration, such as torpor or subnivean life (Webber & McGuire 2022). However, the ca. 100-km fall relocation of Arctic hares on northern Ellesmere Island suggests that in very resource-poor and heterogeneous landscapes, migratory behavior can evolve even in relatively small species. Shedding light on the prevalence, drivers, mechanisms and constraints of Arctic hare seasonal relocations thus offers great opportunities to better understand the evolution of long-distance movements in terrestrial mammals. Of particular interest is the stopover ecology of hares, since the respective roles of energy intake and migration speed are critical to the fitness of migrating animals (Åkesson & Hedenström, 2007; Evans & Bearhop 2022).

Stopover locations of hares were about twice as numerous as traveling locations, suggesting that the time spent at stopovers was also twice the time spent traveling (thus, hares likely spent ca. 67% of their relocating time at stopover sites). This strongly deviates from observations in ungulates such as mule deer (*Odocoileus hemionus*), which maximize energy intake by spending 95% of their migration time at stopover sites (Sawyer & Kauffman 2011). Our observation, however, does roughly fit predictions from the range equation for animal runners published by Hedenström (2003), which predicts potential traveling distance as a function of fuel load. This equation, which is derived from the time minimization migration strategy observed in birds, defines range as a function of diminishing return with respect to added fuel. It suggests that, for running migrants, the optimal ratio between time spent refueling and time spent traveling should be approximately 3:1 (or 75% of migrating time spent at stopover sites). More data from more species and more populations are clearly needed to fully understand time allocation during migration in running animals.

Environmental features selected by hares during their fall relocation suggest that stopovers were partly used to replenish body reserves. Our observation that hares stopped preferentially in patches of heterogeneous vegetation may indeed be related to both their preference for willows and the spacing pattern of willow patches (Klein & Bay 1994), although this needs to be confirmed. Regarding the interaction between vegetation and elevation, this may reflect a potential trade-off between maximization of food acquisition and minimization of predation risk. While the presence of vegetation allows the renewal of energy reserves, high elevation may offer better detection of predators. Intriguingly, low-elevation areas relatively devoid of vegetation were also selected for stopping. This could suggest that hares use stopovers for purposes beyond merely replenishing energy supply. Stopover functions also include providing shelter against adverse weather or predators. In this context, selection of open areas such as frozen lakes may facilitate detection and escape from predators, as observed in woodland caribou (Fortin et al. 2008).

High throughout GPS tracking, currently underway in our study population, will provide more precise, more frequent, and better time-distributed location data for Arctic hares on northern Ellesmere Island, allowing more robust testing of hypotheses. While our study is the first to assess Arctic hare habitat selection in a context of seasonal dynamics, it is also a rare example of detailed investigation of small mammal movement ecology in the polar desert. Such research has never been so important, given the drastic effects that change in permafrost and snow conditions have and will continue to have its effect on tundra wildlife (Berteaux et al. 2016).

### Supplementary Information

Below is the link to the electronic supplementary material.

Supplementary file1 (DOCX 76 KB)

## Data Availability

The locations dataset used in this study is part of the Arctic Animal Movement Archive, is freely available in MoveBank and is stored in the MoveBank Data Repository at https://www.movebank.org/cms/movebank-main.

## Code availability

Code is freely available upon request to the authors.
