# Supplementary material for: Vegetation biomass and topography are associated with seasonal habitat selection and fall translocation behavior in Arctic hares
Source: Oecologia. 2024 Mar 30;204(4):775–88. doi: 10.1007/s00442-024-05534-x (PMC11062897; doi:10.1007/s00442-024-05534-x)

## Electronic Supplemental Material

Vegetation biomass and topography are associated with seasonal habitat selection and fall translocation behavior in Arctic hares

Ludovic Landry-Ducharme, Sandra Lai, François Vézina, Andrew Tam, Dominique Berteaux^*^

******* Corresponding author (email: [dominique_berteaux@uqar.ca](mailto:dominique_berteaux@uqar.ca); phone: +1 418 723 1986, #1910)

**Table S1.** Mean, standard deviation (SD), 95% range, minimum (Min) and maximum (Max) values of continuous variables, as well as proportion of the landscape covered by each level of the qualitative variable (aspect), used to characterize availability areas in analyses of summer and winter habitat selection in Arctic hares on northeastern Ellesmere Island (Nunavut, Canada). See Table 1 for description of variables.

| ***Variable*** | ***Mean ± SD*** | ***95% range*** | ***Min*** | ***Max*** | ***Proportion*** |
| --- | --- | --- | --- | --- | --- |
| *Summer* |  |  |  |  |  |
| SAVI_mean | 0.23 ± 0.20 | 0.00 – 0.73 | -1.49 | 1.50 |  |
| Elevation_mean (m) | 299.58 ± 164.18 | 30.26 – 620.53 | 16.23 | 707.74 |  |
| Slope angle (°) | 6.54 ± 6.65 | 0.56 – 26.90 | 0.00 | 65.65 |  |
| Rugosity | 1.001 ± 0.004 | 1.000 – 1.009 | 1.000 | 1.562 |  |
| North |  |  |  |  | 0.26 |
| East |  |  |  |  | 0.35 |
| South |  |  |  |  | 0.25 |
| West |  |  |  |  | 0.13 |
| *Winter* |  |  |  |  |  |
| SAVI_mean | 0.22 ± 0.24 | -0.04 – 0.74 | -1.50 | 1.50 |  |
| Elevation_mean (m) | 543.99 ± 251.23 | 66.68 – 1053.58 | 5.10 | 1922.10 |  |
| Slope angle (°) | 10.69 ± 9.94 | 0.65 – 36.56 | 0.00 | 87.84 |  |
| Rugosity | 1.00 ± 0.01 | 1 – 1.01 | 1.00 | 15.84 |  |
| North |  |  |  |  | 0.22 |
| East |  |  |  |  | 0.27 |
| South |  |  |  |  | 0.27 |
| West |  |  |  |  | 0.24 |

**Table S2.** Median, mean and standard deviation (SD) of environmental variables selected to analyze summer and winter habitat selection of Arctic hares studied on northeastern Ellesmere Island (Nunavut, Canada). Metrics are based on hares included in each seasonal analysis (Table S4 gives details on individuals selected for analyses). See Table 1 for description of variables.

| *Variable* | *Used home ranges* | | *Available home ranges* | |
| --- | --- | --- | --- | --- |
|  | *Median* | *Mean ± SD* | *Median* | *Mean ± SD* |
| *Summer* |  |  |  |  |
| SAVI_mean | 0.25 | 0.25 ± 0.03 | 0.22 | 0.22 ± 0.06 |
| SAVI_SD | 0.20 | 0.21 ± 0.02 | 0.14 | 0.15 ± 0.05 |
| Elevation_mean (m) | 90.1 | 103.2 ± 26.2 | 382.8 | 354.4 ± 132.2 |
| Rugosity | 1.005 | 1.006 ± 0.003 | 1.007 | 1.009 ± 0.006 |
| Slope_mean (degrees) | 4.6 | 5.0 ± 1.1 | 5.35 | 5.69 ± 1.64 |
| North (proportion) | 0.26 | 0.25 ± 0.5 | 0.26 | 0.25 ± 0.11 |
| East (proportion) | 0.31 | 0.34 ± 0.09 | 0.39 | 0.39 ± 0.14 |
| South (proportion) | 0.23 | 0.24 ± 0.06 | 0.24 | 0.25 ± 0.10 |
| West (proportion) | 0.14 | 0.17 ± 0.08 | 0.10 | 0.11 ± 0.08 |
| *Winter* |  |  |  |  |
| SAVI_mean | 0.50 | 0.46 ± 0.16 | 0.20 | 0.24 ± 0.10 |
| SAVI_SD | 0.26 | 0.25 ± 0.04 | 0.15 | 0.15 ± 0.05 |
| Elevation_mean (m) | 540.3 | 592.8 ± 118.9 | 574.3 | 542.6 ± 167.5 |
| Rugosity | 1.02 | 1.03 ± 0.03 | 1.01 | 1.02 ± 0.02 |
| Slope_mean (degrees) | 7.57 | 10.19 ± 7.16 | 7.54 | 7.97 ± 3.22 |
| North (proportion) | 0.25 | 0.25 ± 0.08 | 0.22 | 0.22 ± 0.07 |
| East (proportion) | 0.20 | 0.21 ± 0.13 | 0.27 | 0.27 ± 0.08 |
| South (proportion) | 0.24 | 0.25 ± 0.09 | 0.25 | 0.26 ± 0.08 |
| West (proportion) | 0.25 | 0.30 ± 0.12 | 0.24 | 0.25 ± 0.10 |

**Table S3.** Median, mean and standard deviation (SD) of environmental variables used in a latent selection difference analysis of stopover and traveling buffered locations during the 2019 fall relocation of 21 Arctic hares on northeastern Ellesmere Island (Nunavut, Canada). When two variables were correlated (Spearman rank correlation r > 0.7), only the most biologically relevant one was kept in the analysis. See Table 1 for description of variables.

| *Variable* | *Stopover* | | *Travel* | |
| --- | --- | --- | --- | --- |
|  | *Median* | *Mean ± SD* | *Median* | *Mean ± SD* |
| SAVI_mean | 0.27 | 0.29 ± 0.14 | 0.23 | 0.27 ± 0.15 |
| SAVI_SD | 0.16 | 0.16 ± 0.07 | 0.13 | 0.14 ± 0.08 |
| Elevation_mean (m) | 590.6 | 531.1 ± 215.6 | 559.7 | 534.5 ± 201.7 |
| Slope_mean (degrees) | 7.45 | 8.58 ± 4.83 | 6.75 | 8.44 ± 5.54 |
| Northness_mean | 0.04 | 0.03 ± 0.48 | -0.03 | -0.02 ± 0.50 |
| Eastness_mean | -0.20 | -0.17 ± 0.47 | -0.14 | -0.10 ± 0.49 |

**Table S4.** Identification code, sex (F = female, M = male), and number of Argos filtered locations for 18 Arctic hares included in summer 2019 or winter 2019-2020 regional-scale habitat selection analyses on northeastern Ellesmere Island (Nunavut, Canada). Only individuals with > 50 locations obtained in summer or winter were retained for the analyses, for a total of 11 individuals in summer and 11 in winter (three individuals are included in both analyses).

| *ID (sex)* | *N summer locations* | *N winter locations* |
| --- | --- | --- |
| BBGG (F) | – | 151 |
| BBYY (F) | – | 123 |
| BGVY (F) | 81 | – |
| BVVY (F) | 80 | – |
| BYBY (M) | – | 83 |
| BYRV (F) | 71 | – |
| GBVV (F) | 73 | – |
| GBYR (F) | 76 | – |
| GGYY (F) | 52 | 54 |
| RGGG (F) | – | 113 |
| RGRG (M) | – | 218 |
| RRYV (F) | 73 | – |
| RVBV (F) | – | 64 |
| RYRY (M) | – | 89 |
| VBRV (F) | 72 | – |
| VRRB (F) | 59 | 68 |
| VVBB (F) | 77 | 186 |
| YVVY (F) | 56 | 192 |
| Mean ± SD | 70 ± 10 | 122 ± 62 |
| N individuals | 11 | 11 |

**Table S5.** Identification code, sex (F = female, M = male), number of Argos filtered locations used in a hidden Markov model, number (percentage) of locations identified as stopover or traveling locations, and ratio between stopover and travel locations, for 21 Arctic hares tracked during their 2019 fall relocation on northeastern Ellesmere Island (Nunavut, Canada).

| *ID (sex)* | *Locations* | *Stopover locations (%)* | *Travel locations (%)* | *Ratio* |
| --- | --- | --- | --- | --- |
| BBYY (F) | 46 | 25 (54) | 21 (46) | 1.2 |
| BGGR (F) | 28 | 20 (71) | 8 (29) | 2.5 |
| BGVY (F) | 52 | 39 (75) | 13 (25) | 3.0 |
| BVVY (F) | 34 | 24 (71) | 10 (29) | 2.4 |
| BYRV (F) | 38 | 19 (50) | 19 (50) | 1.0 |
| BYYG (F) | 17 | 11 (65) | 6 (35) | 1.8 |
| GBVV (F) | 40 | 30 (75) | 10 (25) | 3.0 |
| GBYR (F) | 27 | 18 (67) | 9 (33) | 2.0 |
| GGYY (F) | 14 | 6 (43) | 8 (57) | 0.7 |
| GVRG (F) | 47 | 29 (62) | 18 (38) | 1.6 |
| RBBB (F) | 46 | 29 (63) | 17 (37) | 1.7 |
| RGGG (F) | 48 | 27 (56) | 21(44) | 1.3 |
| RGRG (M) | 35 | 8 (23) | 27 (77) | 3.4 |
| RRYV (F) | 29 | 19 (66) | 10 (34) | 1.9 |
| VBRV (F) | 44 | 34 (77) | 10 (23) | 3.4 |
| VRRB (F) | 41 | 14 (34) | 27 (66) | 0.5 |
| VVBB (F) | 38 | 26 (68) | 12 (32) | 2.2 |
| YGRG (M) | 39 | 30 (77) | 9 (23) | 3.3 |
| YVVY (F) | 35 | 23 (66) | 12 (34) | 1.9 |
| YYRR (F) | 49 | 30 (61) | 19 (39) | 1.6 |
| YYYV (F) | 46 | 31 (67) | 15 (33) | 2.1 |
| Mean ± SD | 37.8 ± 10.2 | 24.3 ± 7.9  (64.0 ± 11.4) | 13.4 ± 5.6  (36.0 ± 11.4) | 2.0 ± 0.9 |

**Table S6.** Coefficient estimate (β), standard error (SE) and 95% confidence interval (95% CI) of variables (including an interaction) retained by the best-fitting model in a latent selection difference analysis of traveling (0) and stopover (1) buffered locations during the 2019 fall relocation of Arctic hares on northeastern Ellesmere Island (Nunavut, Canada). See Table 1 for description of variables.

| *Variable* | *β ± SE* | *95% CI* |
| --- | --- | --- |
| SAVI_mean | 0.08 ± 0.10 | -0.19 – 0.21 |
| SAVI_SD | 0.29 ± 0.10 | 0.09 – 0.48 |
| Elevation_mean | 0.19 ± 0.09 | 0.01 – 0.36 |
| Northness | 0.16 ± 0.08 | -0.01 – 0.33 |
| SAVI_mean * Elevation_mean | 0.35 ± 0.10 | 0.16 – 0.55 |

**Figure S1.** Proportion of behavioral states (travel (red), stopover (blue)) in each Argos isotropic error class (1 = 1,500 m; 2 = 500 m; 3 = 250 m; A = no error estimation) associated with locations recorded during the 2019 fall relocation of 21 Arctic hares on northeastern Ellesmere Island (Nunavut, Canada). The proportion of behavioral states does not differ significantly in any of the Argos error classes (Chi squared test: χ² = 6.77, d.f. = 3, p-value = 0.08)


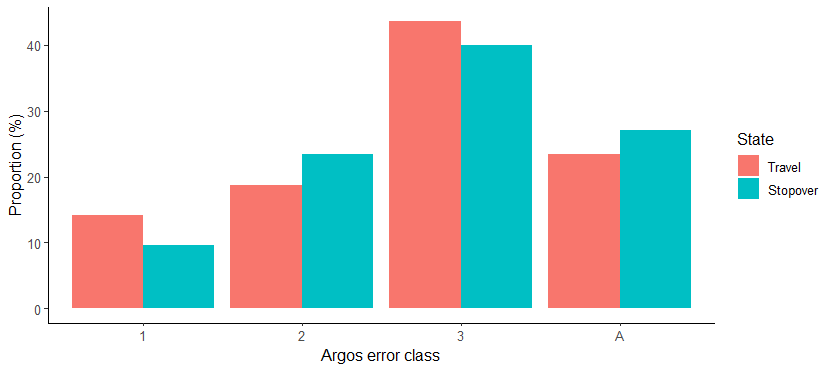

Supplement: Supplementary file 1 — Supplementary file1 (DOCX 76 KB) [file 442_2024_5534_MOESM1_ESM.docx]
